# Treatment of obstructive sleep apnea using a custom-made titratable duobloc oral appliance: a prospective clinical study

**DOI:** 10.1007/s11325-012-0721-3

**Published:** 2012-05-13

**Authors:** M. Dieltjens, O. M. Vanderveken, E. Hamans, J. A. Verbraecken, K. Wouters, M. Willemen, W. A. De Backer, P. H. Van de Heyning, M. J. Braem

**Affiliations:** 1 Department of Special Care Dentistry, Antwerp University Hospital, Wilrijkstraat 10, 2650 Edegem, Antwerp Belgium; 2 ENT Department and Head and Neck Surgery, Antwerp University Hospital, Wilrijkstraat 10, 2650 Edegem, Antwerp Belgium; 3 Multidisciplinary Sleep Disorders Centre, Antwerp University Hospital, Wilrijkstraat 10, 2650 Edegem, Antwerp Belgium; 4 Department of Pulmonary Medicine, Antwerp University Hospital, Wilrijkstraat 10, 2650 Edegem, Antwerp Belgium; 5 Department of Scientific Coordination and Biostatistics, Antwerp University Hospital, Wilrijkstraat 10, 2650 Edegem, Antwerp Belgium; 6 Faculty of Medicine and Health Sciences, University of Antwerp, Wilrijkstraat 10, 2650 Edegem, Antwerp Belgium

**Keywords:** Snoring, Sleep-disordered breathing, Daytime sleepiness, Mandibular repositioning appliance

## Abstract

**Purpose:**

This prospective clinical study investigates the efficacy of a specific custom-made titratable mandibular advancement device (MAD) for the treatment of obstructive sleep apnea (OSA). This MAD has attachments in the frontal teeth area that allow for progressive titration of the mandible.

**Methods:**

Sixty-one adult OSA patients were included (age, 46.7 ± 9.0 years; male/female ratio, 45/16; apnea–hypopnea index (AHI), 23.2 ± 15.4 events/h sleep; body mass index, 27.9 ± 4.1 kg/m²). After an adaptation period, titration started based on a protocol of symptomatic benefit or upon reaching the physiological limits of protrusion. As a primary outcome, treatment response was defined as an objective reduction in AHI following MAD treatment of ≥50 % compared to baseline, and treatment success as a reduction in AHI with MAD to less than 5 and 10 events/h sleep. Compliance failure was defined as an inability to continue treatment.

**Results:**

A statistically significant decrease was observed in AHI, from 23.4 ± 15.7 at baseline to 8.9 ± 8.6 events/h with MAD (*p* < 0.01). Treatment response was achieved in 42 out of 61 patients (68.8 %), whereas 42.6 % met criteria of AHI < 5 and 63.9 % achieved an AHI < 10 events/h sleep, respectively. Four patients (6.6 %) were considered as “compliance failures.”

**Conclusions:**

The present study has evaluated the efficacy of a specific custom-made titratable MAD in terms of sleep apnea reduction.

## Introduction

Obstructive sleep apnea (OSA) is a highly prevalent public health issue affecting an estimated 2 % of middle-aged women and 4 % of middle-aged men [1]. OSA is characterized by recurrent apneas or hypopneas during sleep, resulting in hypoxemia, arousal from sleep, and sleep fragmentation [2, 3]. OSA is an independent risk factor for cardiovascular complications and can be associated with a relatively high morbidity and mortality [1, 4, 5]. Continuous positive airway pressure (CPAP) is regarded as the gold standard treatment for OSA [6]. However, despite its high therapeutic efficacy, it is not always well tolerated by patients, resulting in a limited clinical effectiveness [7, 8]. Oral appliances are considered a noninvasive treatment option for patients with mild to moderate sleep apnea and for patients who do not comply with or refuse CPAP treatment [9, 10]. Mandibular advancement devices (MADs), worn intraorally at night in order to advance the mandible and to reduce the collapsibility of the upper airway, are the most common class of oral appliances used to treat OSA [8, 10–12]. MAD therapy has been shown to reduce the severity of OSA to a lesser or similar degree than CPAP [8, 13–15]. Currently, there is growing evidence that both CPAP and MADs have a beneficial effect on OSA-related adverse health consequences like cardiovascular morbidity [4, 14, 16–18].

There is a huge variety of commercially available MADs each with its particular concept [12, 19, 20]. Oral appliance design has been suggested as one of the factors that will affect treatment success, adherence, and side effects [19, 20]. Prefabricated MADs are made out of thermoplastic material, the “boil-and-bite devices”, and can be fitted chair-sided, preferably by a dental sleep professional at the outpatient clinic. However, research showed that this type of MADs has limited effectiveness [12, 21, 22]. Custom-made MADs are made in a dental technical lab from individual impressions of a patient’s tooth arches [11, 21]. It is found that custom-made MADs are better tolerated and provide a higher efficacy compared to thermoplastic appliances [16, 21]. The MAD was originally available as a “monobloc” type with the upper and lower parts statically connected and has evolved into the current “duobloc” or “titratable” generation with separate upper and lower parts that are dynamically interconnected. This allows for gradual forward positioning of the mandible until the optimal mandibular position is reached within the patients’ physiological protrusive limits. This gradual positioning of the mandible, called the titration process, is a straightforward mechanism, but the procedure is complex because of the lack of a gold standard method [23]. Titratable MADs allow for easy protrusive changes without the need for corrective work in the dental laboratory as is generally the case with the monobloc MAD that should require additional protrusive adjustment.

Last generation MADs can be divided in a group of devices that cover all teeth in the upper and lower jaw versus a group that relies on selective embracement of tooth arc areas. The MAD used in this study belongs to the latter group, consisting of an upper and lower jaw clip, attached to each other via a titration screw mechanism located in the frontal teeth area allowing for precise adjustment of mandibular protrusion as well as adjustment of the amount of vertical opening. This prospective clinical study assesses the efficacy of this specific custom-made MAD with reduction of sleep apnea as the main outcome.

## Materials and methods

### Study population

All patients in this prospective clinical study were diagnosed with OSA based on a recent polysomnography, with an apnea–hypopnea index (AHI) of greater than 5 events/h of sleep [2]. A standard ear, nose, and throat (ENT) clinical examination was performed in all patients and included a detailed clinical history, clinical exploration of ear, nose, and throat with examination of pharyngeal and laryngeal findings (webbing, size of uvula, soft palate, and tonsils). On the basis of this ENT examination, the patient was considered a suitable candidate for MAD treatment and for this clinical study. Patients were then referred to the dental sleep professional, who made a full dental examination including orthopantography. Exclusion criteria were mainly dental: patients were not judged suitable for MAD treatment if they suffered from any pre-existing active temporomandibular joint dysfunction, if their dental status or periodontal health precluded them from wearing an oral appliance, or if they were fully edentulous [21, 24].

Sixty-one consecutive adult OSA patients (age, 46.7 ± 9.0 years; male/female ratio, 45/16; AHI, 23.2 ± 15.4 events/hour of sleep; body mass index (BMI), 27.9 ± 4.1 kg/m²) were included after written informed consent was provided. Their age ranged from 26 to 70 years, with a mean age of 46.7 years. Baseline characteristics of the study population are presented in Table 1. They started MAD therapy between 1st January 2009 and 1st January 2010.

**Table 1:** Baseline characteristics of the study population

Parameter	Mean ± SD
Age (years)	46.7 ± 9.0
Gender	73.8 % male
Body mass index, BMI (kg/m²)	27.9 ± 4.1
Weight (kg)	85.2 ± 15.7
Apnea–hypopnea index, AHI (events/h)	23.2 ± 15.4
Epworth Sleepiness Scale, ESS (0–24)	10 ± 5
Visual Analogue Scale for snoring, VAS (0–10)	7 ± 3

Data are expressed as mean ± standard deviation (SD) or percentages

Ethical approval for this study was obtained from institutional review boards of the Antwerp University Hospital (Belgian registration number: B30020110946).

### The titratable mandibular advancement device

**Fig. 1:**
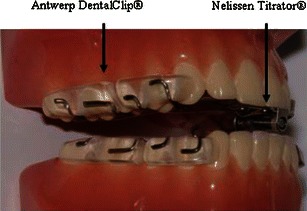
The RespiDent Butterfly® MAD, consisting of two clips (Antwerp Dental Clip®), attached to each other allowing adjustment of the mandibular protrusion in the frontal teeth area, as well as vertical positioning

A custom-made, titratable, commercially available duobloc MAD with an interconnecting mechanism located in the frontal teeth area allowing precise adjustment of mandibular protrusion was selected (RespiDent Butterfly® MRA, Orthodontic Clinics NV, Antwerp, Belgium). The appliance consists of two clips (Antwerp DentalClip®) that are attached to each other via a small screw system located in the frontal teeth area (Nelissen Titrator®) (Fig. 1). Both clips are interconnected using the advancement screw, which is mounted horizontally through the O-ring of the vertical screw of the clip on the lower jaw up and extending to the eyelet mounted on the clip in the upper jaw. A safety elastic will prevent this screw from sliding out the O-ring on unwanted disconnection from the eyelet. The advancement of the mandible can be titrated gradually by giving a 360 ° clockwise turn of the horizontal screw, thereby advancing the mandible 0.5 mm (Fig. 1). The vertical opening of the MAD, being the distance between the incisal edges of the upper and lower incisors, can be adjusted by turning the vertical screw counterclockwise. However, for this study, it was set as low as possible and patients were instructed not to change it. The MAD itself “snaps” on the tooth arches due to the elasticity provided in the appliance by the stainless steel wires that connect the vestibular and lingual acrylic embracing parts. Prior to therapy, the maximal protrusion was measured three times in each patient and averaged. A dental registration bite allowed for the transfer of the selected starting protrusion, being 50 % of the maximal protrusion, into the MAD via the horizontal screw. The fitting as well as the protrusive start position of the MAD was checked by a qualified dental sleep professional. The patients were instructed to titrate every 3 days after three to four nights of habituation to the appliance, until either improvement or resolution of symptoms like snoring and/or excessive daytime sleepiness was achieved or until the physiological protrusive limit for the individual patient was reached.

### Objective measurement: polysomnography

Overnight polysomnography (PSG) included the recording of electroencephalography, right and left electrooculography, electromyography of the genioglossus and tibialis anterior muscle, electrocardiography, and oxygen saturation using pulse oximetry with a finger probe. Respiratory variables were recorded, including nasal airflow by means of an external thermistor and nasal pressure by using a nasal pressure cannula. Respiratory effort was measured using respiratory inductance plethysmography. A microphone records snoring qualitatively and body position is monitored using a piëzo-electrical sensor.

The sleep recordings were stored on a computer system (Brain RT version 1/patch pack 2, OSG, Rumst, Belgium) and were manually scored thereafter in a standard way by a qualified sleep technician [2, 25]. An apnea was defined as a total cessation of airflow during at least 10 s. A hypopnea was defined as a decrease of airflow of at least 50 % from the preceding stable airflow in the last 2 min, or a clear decrease in airflow associated with an oxygen desaturation of at least 4 % and/or an arousal, during at least 10 s. The AHI is the average number of apneas and hypopneas per hour of sleep. Based on the AHI, the following levels of severity are defined: mild sleep apnea with a score of 5 < AHI ≤ 15, moderate sleep apnea scored 15 < AHI ≤ 30, and severe sleep apnea with AHI > 30 events/h of sleep [2].

All patients in this prospective clinical study underwent two overnight polysomnographic recordings: the first PSG was performed to diagnose their sleep-related breathing disorder and will be referred to as the baseline recording. Once the MAD was optimally titrated relative to resolution of symptoms or reaching the maximum comfortable limit, the patient was referred to the sleep laboratory for a follow-up polysomnography with the MAD in situ. Doing so, the therapeutic effect of the MAD at the titrated position could be objectively assessed by comparing the baseline polysomnography without MAD with the follow-up PSG with MAD. The time lag between fitting of the MAD and the follow-up PSG with MAD in situ was on average 4 ± 3 months.

### Subjective measurements: Epworth Sleepiness Scale and Visual Analogue Scale for snoring

#### Epworth Sleepiness Scale (ESS)

The degree of daytime sleepiness can be assessed using a self-administered questionnaire, called the Epworth Sleepiness Scale [26]. The ESS is a measure of the probability of falling asleep in a variety of day life situations. The patients were asked to rate his or her probability of falling asleep or dozing off on a scale of 0 to 3 for eight different situations. The ESS score is the sum of the eight item scores and can range from 0 to 24. Classically, an ESS score higher than 10/24 defines the presence of excessive daytime sleepiness [26]. Twenty-seven patients (50.9 %) suffered from excessive daytime sleepiness (ESS > 10/24) before starting MAD treatment.

#### Visual Analogue Scale (VAS) for snoring

A standard 10-point VAS ranging from 0 to 10 with 0 equaling no snoring and 10 causing the bed partner to leave the room or sleep separately was used to evaluate the subjective status of snoring, as assessed by the bed partner [27]. Heavy snoring was defined as a VAS snoring index of at least 7. A decrease of at least three points on VAS for snoring during treatment with MAD compared to baseline represents a decrease of one level of snoring severity and was therefore considered “satisfactory” [21]. To be considered as an “important” reduction, snoring had to be reduced to a snoring index that was no longer evaluated as bothersome, i.e., to a snoring index of ≤3 [21].

### Treatment outcome measures

Prior to the start of the study, the following decisions were made as to the treatment outcomes.

#### Primary outcome

“Responders” were defined as patients with a reduction in AHI following MAD treatment of ≥50 % compared to baseline, and “non-responders” were defined as patients with a reduction in AHI of <50 % [28]. Treatment success was defined as a reduction of the AHI to less than 5 or 10/h of sleep with MAD. Compliance failure was defined as an inability of the patient to continue treatment for whatever reason mentioned by the patient.

#### Secondary outcomes

For the subjective secondary outcomes, questionnaires filled out at baseline and at the time of the follow-up PSG were analyzed. The VAS for snoring was assessed in the 45 patients who had a bed partner that was able to report the effect on snoring. To assess patient satisfaction with the MAD therapy, patients were asked to score their therapy appreciation at the follow-up visits with 0/10 equaling “very bad” and 10/10 equaling “very satisfied.” This evaluation was done at the scheduled follow-up sessions and at the time of the follow-up PSG.

Patient subjective compliance was assessed at the scheduled follow-up visits using a questionnaire, with regard to how many nights per week the MAD was worn and for how many hours per night. Regular use was defined by at least 4 h of MAD use on 70 % of the days monitored [29].

#### Combined primary and secondary outcomes

Success definition including combining primary outcome with symptomatic reduction in snoring and/or excessive daytime sleepiness was also included. Complete response was defined as an “important” reduction of snoring without excessive daytime sleepiness (ESS < 11/24) plus a decrease in AHI to <5 events/h. Partial response was defined as a “satisfactory” decrease of snoring without excessive daytime sleepiness (ESS < 11/24) plus a 50 % or greater reduction in AHI. Treatment failure was defined as ongoing clinical symptoms and/or a less than 50 % reduction in AHI.

### Statistical analysis

Data were statistically analyzed using SPSS software (SPSS version 17.0, Statistical Package for Social Sciences, SPSS Inc., Chicago, USA). Normality of distribution was assessed using QQ plots. All data were assessed as not normal distributed. Nonparametric Wilcoxon-signed rank test for paired observations was performed to test the evolution of the different variables. The significance level was set at 5 %, *p* < 0.05 was considered significant.

## Results

### Study population

Polysomnographic re-evaluation with the MAD in situ was obtained in 57 out of 61 patients: 4 out of the 61 patients (6.6 %) did not continue their treatment because of teeth discomfort or pain, excessive salivation, and/or muscle tenderness. Weight and BMI of the patients did not change significantly during MAD therapy.

### MAD effect on outcome measures

#### Primary outcome

**Fig. 2:**
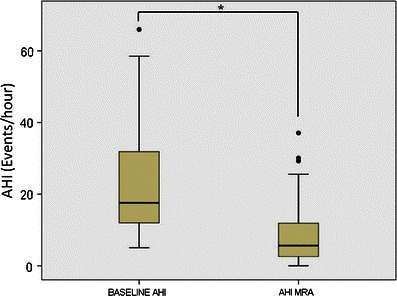
Evolution of the apnea–hypopnea index (AHI). *Bullets* outliers; *p < 0.05

At the follow-up polysomnography with the MAD in situ, a statistically significant improvement was observed regarding AHI. AHI values decreased from 23.4 ± 15.7 events/h at baseline to 8.9 ± 8.6 events/h with MAD (*p* < 0.01) (Fig. 2). In four patients (6.6 %), deterioration in apnea severity was noticed after MAD treatment.

The different success definitions with their respective percentages are summarized in Table 2. According to the above stated criteria, 42 out of 61 patients (68.8 %) were found to be “responders” with a reduction in AHI of ≥50 % following MAD treatment compared to baseline, and 15 patients reacted as “non-responders” with reduction in AHI of <50 %. Twenty-six patients (42.6 %) met criteria of treatment success defined as an AHI < 5/h with MAD in situ, and 39 patients (63.9 %) achieved an AHI < 10/h with MAD in situ. More strict criteria of treatment success were also analyzed: 25 patients (41.0 %) fulfilled success criteria defined as a reduction in AHI of ≥50 % and AHI < 5/h, whereas in 34 patients (55.7 %), AHI reduced with ≥50 % and AHI < 10/h with MAD in situ. Four out of 61 patients (6.6 %), two patients with mild sleep apnea and two patients diagnosed with severe sleep apnea, could not tolerate the MAD and did not continue their treatment and were defined as “compliance failures.”

**Table 2:** Success definitions and results in the present study

Success definition	Percentage (%)
Treatment response (decrease of ≥50 % in AHI)	68.8
Treatment success (AHI < 5/h)	42.6
Treatment success (AHI < 10/h)	63.9
Decrease of ≥50 % in AHI and AHI < 5/h	41.0
Decrease of ≥50 % in AHI and AHI < 10/h	55.7
Compliance failure	6.6

The number of patients in each category of severity decreased as a result of the MAD treatment and an overall reduction in the number of patients with OSA was noted, decreasing from 57 to 31 patients. Responder rates, defined as a reduction in AHI with MAD of ≥50 % compared to baseline showed a comparable pattern in mild, moderate, and severe sleep apnea categories with 66.7 % (16 out of 24 patients), 72.2 % (13 out of 18 patients), and 68.4 % (13 out of 19 patients), respectively. Success rates for AHI < 5/h according to these severity categories were 66.7 % (16 out of 24 patients), 33.3 % (6 out of 18 patients) and 22.2 % (4 out of 18 patients) for mild, moderate, and severe sleep apnea, respectively, and for AHI < 10/h, we found success percentages of 83.3 % (20 out of 24 patients) for mild sleep apnea patients, 66.7 % (12 out of 18 patients) for moderate sleep apnea patients, and 38.9 % (7 out of 18 patients) for severe sleep apnea.

#### Secondary outcomes

##### Epworth Sleepiness Scale

A statistically significant (*p* < 0.01) decrease in daytime sleepiness was observed (*n* = 53) following MAD treatment, with an ESS at baseline of 10 ± 5 compared to 7 ± 4 with MAD. Twenty-seven patients (50.9 %) suffered from excessive daytime sleepiness (ESS > 10/24) before starting MAD treatment. In 63.0 % of these patients, the ESS lowered to a score of ≤10/24, suggesting adequate resolution of excessive daytime sleepiness following MAD treatment.

##### Visual Analogue Scale for snoring

A statistically significant decrease in VAS for snoring was observed in all 45 patients who had a bed partner that was able to report this effect. VAS decreased from 7 ± 3 at baseline to 3 ± 2 at follow up PSG (*p* < 0.01). Twenty-eight patients were defined as heavy snorers, with a VAS snoring index of at least 7 prior to treatment. In 20 of these 28 heavy snorers (71.4 %), the subjective score for snoring was reduced to a level below 7. In general, a decrease of at least three points on VAS for snoring was noted in 28 out of 45 patients (62.2 %), while 93.3 % of patients reported a reduction in snoring to a VAS of ≤3 with MAD.

##### Patient satisfaction

On average, patients give their therapy satisfaction a score of 8/10 (range 4–10). Only one patient scored the therapy satisfaction below a score of 5/10 because of a reported pain in the temporomandibular joint.

##### Subjective compliance

Patients reported the use of their MAD being on average 7 ± 1 nights per week. Forty-eight patients (87.2 %) claimed to use their MAD every night. Four patients claimed to use their MAD six nights a week, two patients were wearing it five nights a week, and one patient used it only three nights a week. On average, patients reported using the MAD for 7 ± 1 h per night, with a minimum of 4 h. Fifty-four out of 56 patients (96.4 %) used the MAD for at least 4 h on 70 % of the days monitored and met the criteria for regular use.

#### Combined primary and secondary outcomes

The evolution of objective and subjective outcome measures is summarized in Table 3. It was found that the objective as well as the subjective variables showed a parallel shift towards less severe OSA in all severity subgroups following MAD treatment.

**Table 3:** Evolution of primary and secondary outcome measures according to OSA severity

OSA severity (variables)	Baseline PSG (without MAD)	Follow-up PSG (with MAD)	*p* value
Mild OSA			
AHI	10.0 ± 3.0	4.7 ± 6.2	0.001
VAS	7 ± 3	3 ± 2	<0.001
ESS	9 ± 5	8 ± 4	0.048
Moderate OSA			
AHI	20.4 ± 3.6	8.8 ± 6.1	<0.001
VAS	7 ± 2	2 ± 1	0.001
ESS	10 ± 5	8 ± 5	0.023
Severe OSA			
AHI	44.2 ± 11.0	14.5 ± 10.6	<0.001
VAS	7 ± 3	3 ± 3	0.009
ESS	11 ± 5	7 ± 5	0.004
All patients			
AHI	23.4 ± 15.7	8.9 ± 8.6	<0.001
VAS	7 ± 3	3 ± 3	<0.001
ESS	10 ± 5	7 ± 4	<0.001

Success definition including symptomatic reduction in snoring and/or excessive daytime sleepiness was also included: complete response was reached in 19 patients (31.1 %), 11 patients (18.0 %) were defined as partial responders, and 27 patients (44.3 %) were defined as treatment failure.

## Discussion

This prospective clinical study has evaluated a specific custom-made, titratable MAD with attachments in the frontal teeth area that allow for progressive titration of the mandible. The data demonstrate that this MAD is effective in treating obstructive sleep apnea by significantly improving the AHI, subjective snoring, and daytime sleepiness in most patients diagnosed with OSA.

In the majority of patients (42 out of 61 patients; 68.8 %), the AHI reduces with ≥50 % with MAD compared to baseline. This is a response rate comparable to other studies in the literature where in general 65 % of the patients are defined as responders, with a ≥50 % reduction in AHI with the MAD [13]. In the present study, only 4 patients out of 61 patients (6.6 %) were unable to tolerate the MAD and were defined as compliance failures. This is nevertheless a high acceptance rate, especially when compared with CPAP, which is highly efficacious but associated with a relatively low compliance and acceptance rate [7, 8]: approximately 20 to 40 % will discontinue CPAP treatment after 3 months [30]. It must, however, be mentioned that the present MAD study describes relatively short-term results with a mean follow-up period of 4 ± 3 months. The compliance in continuing MAD users could be different at long-term follow-up, but these data are not yet available.

At present, there is no universal agreement upon definition of a successful treatment outcome with MAD for obstructive sleep apnea [13]. This is illustrated by studies that defined treatment success as a reduction in AHI to below five, 10, or even 20 events/h of sleep, with or without relief of concomitant subjective symptoms [13]. In the present study, treatment success defined as an AHI < 5/h with MAD in situ was achieved in 42.6 and 63.9 % of patients showing an AHI < 10/h with MAD in situ. These are also comparable high success rates in agreement with other studies that reported an average of 42 and 52 % reaching an AHI < 5 or AHI < 10, respectively [13].

The goal of MAD therapy is not only to lower the AHI, but also to relieve concomitant subjective symptoms in order to provide a long-term acceptable therapy [21, 31]. Excessive daytime sleepiness and snoring can be socially disruptive and are often the main complaints of patients and/or bed partners, respectively. In the present study, 50.9 % of the patients (27 out of 53) complained of excessive daytime sleepiness (ESS > 10/24), with 11 out of the 15 women (73.3 %) and 16 out of 38 men (42.1 %) showing a higher prevalence of hypersomnolence among women. In our study, in 17 out of the 27 patients (63.0 %) suffering from excessive daytime sleepiness (ESS > 10/24) at baseline, the ESS lowered to a score of ≤10, suggesting a decrease of ESS with the MAD to levels considered as non-pathological.

The efficacy of MAD in the treatment of patients with severe OSA has been reported in previous studies [32–34]. The present study also demonstrates that the selected MAD is effective in reducing severity of OSA in most of the patients with severe OSA (Table 3). Even more, four patients (23.5 %) with severe OSA who did continue treatment were effectively treated with a final AHI below 5/h sleep with MAD. The success rates, being a reduction in AHI with MAD of ≥50 % compared to baseline and continuing treatment, are relatively high with the MAD used in this study, even for moderate and severe OSA, with 72.2 % for mild OSA and 68.4 % for severe OSA. Despite these encouraging findings, about one third of patients with mild and/or severe OSA are still not adequately treated, indicating that CPAP should be considered a first choice of treatment in those patients [9].

One specific characteristic of this specific MAD is its ability to change the vertical opening. Although any oral appliance will need some interincisal space due to material requirements, it is still controversial whether an increased vertical opening could be beneficial for therapy or not [13, 35–37]. It is suggested that raising the bite might be associated with an aggravation of OSA for some, but not for all OSA patients [38]. Recent findings by Vroegop et al. [39] have furthermore emphasized that increasing vertical opening generally tends to result in a negative effect on the collapsibility of the tongue base. Therefore, the vertical opening was set as low as possible during this study and the patients were instructed not to change it in order to investigate independently the efficacy of the MAD of this controversial topic.

Up to this date, prediction of treatment success with a given MAD is still not possible. Furthermore, it has been reported that the type of oral appliance is one of the factors that will affect treatment success [19, 20]. Until valid prediction becomes available, evaluation of commercially available designs of MADs in a clinical environment will offer a valuable way of studying their power in the treatment of OSA.

## Conclusion

The specific type of custom-made titratable MAD evaluated allows for a quantifiable titration in both horizontal and vertical dimensions, of which only the horizontal component was explored in the present study. The present results indicate that within the limits of the present study, this specific MAD is effective in reducing sleep apnea by lowering the AHI, reducing snoring and excessive daytime sleepiness in a significant way. The MAD has been well tolerated by patients. Future research can now focus on the possibilities of having titration options in both vertical and horizontal dimensions.
